# The “Product of a Village”: FDA as a Partner in Evidence Generation

**DOI:** 10.1016/j.jscai.2022.100561

**Published:** 2023-01-02

**Authors:** Megan Coylewright, Emily P. Zeitler

**Affiliations:** a The Erlanger Heart Institute, Chattanooga, Tennessee; b Dartmouth Health and The Dartmouth Institute, Lebanon, New Hampshire

**Keywords:** Atrial fibrillation, left atrial appendage occlusion, regulatory, sex

*“So many have contributed to my journey, it would be all too simple to deem my career a single effort. It is not. I am a product of a village.” Tina Thompson, 9-time WNBA All-Star and 4-time league champion*

Regulators at the US Food and Drug Administration (FDA) have increasingly sought collaboration with physicians, scientists, academia, industry, and, most notably, patients over the past 10 years. Furthermore, the formal guidance from the FDA communicates priorities as including assurance that they “provide consumers, patients, their caregivers, and providers with understandable and accessible science-based information about the products we oversee” and that the public they seek to protect is well-represented in populations studied in regulatory studies for medical devices. In this effort, they have been doggedly committed.

In this issue of the *JSCAI*, we see a reflection of that commitment in a manuscript by Jiang et al,^1^ a group of FDA regulator-scientists, which addresses ongoing questions of comparative safety and effectiveness of left atrial appendage occlusion (LAAO) compared with those of warfarin anticoagulation. These questions linger among sex subgroups despite previous work using data from registries, claims, and clinical trials.^2^, ^3^, ^4^ In particular, by combining patient-level data from 2 clinical trials^5^^,^^6^ and 2 continuing access protocols,^7^^,^^8^ this analysis improves the power to evaluate sex-specific outcomes from the early WATCHMAN experience that included 30% to 39% of women despite having a similar lifelong risk of atrial fibrillation (AF) compared with men. With these data sets combined, the experience of 754 women is reported, including 646 who underwent implantation of WATCHMAN 2.5.

Among pooled randomized patients, when compared with those treated with warfarin, the authors found a statistically significant reduction in hemorrhagic stroke among men treated with LAAO (adjusted hazard ratio, 0.163; 95% CI, 0.045-0.593; *P* <.05) but not among women treated with LAAO (adjusted hazard ratio, 0.319; 95% CI, 0.053-1.923; *P* =.21). When all 4 data sets were combined and stratified by sex in a time-to-event analysis, warfarin was associated with a significant increase in the risk of hemorrhagic stroke among men and women (*P* <.05 for both). There were no significant differences seen in ischemic stroke or all-cause mortality between LAAO and warfarin in the pooled group of men or women.

Compared with previous work, this research focuses on sex-based outcomes in a manner that is actionable at the bedside by asking: What are the risks and benefits of LAAO versus warfarin for the patient in front of me? When engaging patients in shared decision-making, as is a requirement for LAAO coverage by the Centers for Medicare and Medicaid Services, the patient-centered comparison is not between a patient who is a woman and a patient who is a man. Rather, the comparison of interest is between LAAO and anticoagulation for a specific patient based on their specific characteristics—including sex. On the other hand, comparisons between men and women are valuable when targeting quality improvement initiatives, such as safe femoral access strategies for smaller patients, but are less useful in patient-centered decision-making.

This work does not ask the following question: How can AF-related stroke risk be maximally reduced? The process of answering this question is ongoing and incorporates considerations across cardiovascular medicine and surgery, but in most cases, includes anticoagulation regardless of other interventions, such as surgical LAAO^9^ or AF suppression with ablation^10^ or antiarrhythmics.^11^ As the authors note, their work was limited by the historical nature of the data because comparisons between device-based and drug-based AF-related stroke reduction that informs shared decision-making will vary with increasing use of direct-acting oral anticoagulants and newer versions of LAAO devices among other changes. Importantly, these evolutions likely affect sex subgroups differently; for example, women have a greater background risk of stroke at older ages. Hence, similar to as was performed in this analysis, the medical device ecosystem—including regulators—should be encouraged to examine and reexamine extant data sources for changing signals of risk and benefit. This process is particularly efficient when the data examined are repurposed from previous regulatory investigations, as in this case.

The Center for Devices and Radiological Health has been a leader in Patient Preference Information in research efforts, particularly efforts pertaining to “preference-sensitive decisions” in which there is not one choice that is superior to others; the evidence supporting one option over another varies; and patient preferences are distinct across diverse groups.^12^ Recommendations for performing this kind of research include engaging patients early and often in meaningful ways, including scientific question generation, selection of comparator groups and outcomes, and in framing trial results. When considering options for reducing AF-related stroke risk as part of shared decision-making, best practices include using an evidence-based decision aid to elicit patient goals and values information, and data, such as those from the study by Jiang et al,^1^ help address gaps in knowledge to better inform these preference-sensitive decisions.

Finally, in addition to work like this that harnesses the power of a regulatory body to combine patient-level data to address unanswered questions in major subgroups, it is important to recognize how the efforts at The Center for Devices and Radiological Health have increased the focus on reaching diverse patient populations in clinical trials. For example, the FDA has released a draft document on Diversity in Clinical Trials for public comment.^13^ This effort, and the above-described data, in addition to the manuscript centering data on decision-making that is relevant to patients, is laudable and highlights the opportunities for synergy among FDA regulators, clinicians, investigators, and cardiovascular device manufacturers. In this way, the care we provide at the bedside is truly “the product of a village.”
